# Supplementary material for: Patterns of Focal Amyloid Deposition Using 18F-Florbetaben PET in Patients with Cognitive Impairment
Source: Diagnostics (Basel). 2022 May 31;12(6):1357. doi: 10.3390/diagnostics12061357 (PMC9221882; doi:10.3390/diagnostics12061357)
Supplement: Supplementary file 1 [file diagnostics-12-01357-s001.zip › diagnostics-1739990-supplementary.pdf]

Supplementary Data S1. The calculation procedure and method of deriving conditional probability (CP) employed in this investigation.

1. The posterior cingulate/precuneus and the lateral temporal cortex.

PP \ F	A (-)	A (+)
A (-)	7	12
A (+)	29	12

***P* = 0.013**

P(P+/LT-) = 29/36 = 0.81

P(LT+/P-) = 12/19 = 0.63

Amyloid spreading can occur in order from to the posterior cingulate/precuneus to the lateral temporal cortex.

2. The lateral temporal and parietal cortices.

LT \ P	A (-)	A (+)
A (-)	25	11
A (+)	14	10

***P* = 0.013**

P(LT+/P-) = 14/39 = 0.36

P(P+/LT-) =11/36 = 0.31

Amyloid spreading can occur in order from the lateral temporal cortex to the parietal cortex.

3. The parietal and frontal cortices.

P \ F	A (-)	A (+)
A (-)	32	7
A (+)	19	2

P = 0.097

P(P+/F-) = 19/51 = 0.37

P(F+/P-) = 7/39 = 0.18

4. The frontal and occipital cortices.

F \ O	A (-)	A (+)
A (-)	43	8
A (+)	9	0

P = 0.164

P(F+/O-) = 9/52 = 0.17

P(O+/F-) = 8/51 = 0.16

5. The occipital cortex and the anterior striata.

O \ S	A (-)	A (+)
A (-)	51	1
A (+)	7	1

P = 0.155

P(O+/S-) = 7/58 = 0.12

P(S+/O-) = 1/52 = 0.02

6. The posterior cingulate/precuneus and the parietal cortex.

PP \ P	A (-)	A (+)
A (-)	11	8
A (+)	28	13

P = 0.081

P(PP+/P-) = 28/39 = 0.72

P(P+/PP-) = 8/19 = 0.42

7. The posterior cingulate/precuneus and the frontal cortex.

PP \ F	A (-)	A (+)
A (-)	16	3
A (+)	35	6

$P = 0.008$

P(PP+/F-) = 35/51 = 0.69

P(F+/PP-) = 8/19 = 0.42

Amyloid spreading can occur in order from the posterior cingulate/precuneus to the frontal cortex.

8. The posterior cingulate/precuneus and the occipital.

PP \ O	A (-)	A (+)
A (-)	13	6
A (+)	39	2

P = 0.18

P(PP+/O-) = 39/52 = 0.75

P(O+/PP-) = 6/19 = 0.32

9. The posterior cingulate and the anterior striatum.

PP \ S	A (-)	A (+)
A (-)	17	2
A (+)	41	0

P = 0.68

P(PP+/S-) = 41/58 = 0.71

P(S+/PP-) = 2/19 = 0.11

10. The lateral temporal and frontal cortices.

LT \ F	A (-)	A (+)
A (-)	28	8
A (+)	23	1

P = 0.20

P(LT+/F-) = 23/51 = 0.45

P(F+/LT-) =8/36 = 0.22

11. The lateral temporal and occipital cortices.

LT \ O	A (-)	A (+)
A (-)	33	3
A (+)	19	5

P = 0.14

P(LT+/O-) = 19/52 = 0.37

P(O+/LT-) =3/36 = 0.83

12. The lateral temporal and anterior striata.

LT \ S	A (-)	A (+)
A (-)	35	1
A (+)	23	1

P = 0.016

P(LT+/S-) = 23/58 = 0.40

P(S+/LT-) =1/36 = 0.03

Amyloid spreading can occur in order from the lateral temporal to the anterior striatum.

13. The parietal and occipital cortices.

P \ O	A (-)	A (+)
A (-)	34	5
A (+)	18	3

P = 0.017

P(P+/O-) = 18/52 = 0.35

P(O+/P-) = 5/39 = 0.13

Amyloid spreading can occur in order from the parietal to the occipital cortex.

14. The parietal and anterior striata.

P \ S	A (-)	A (+)
A (-)	38	1
A (+)	20	1

P = 0.028

P(P+/S-) = 20/58 = 0.34

P(S+/P-) = 1/39 = 0.03

Amyloid spreading can occur in order from the parietal to the anterior striatum.

15. The frontal and anterior striata.

F \ S	A (-)	A (+)
A (-)	50	1
A (+)	8	1

P = 0.135

P(F+/S-) = 8/58 = 0.14

P(S+/F-) = 1/51 = 0.02
